# Surgery, adjuvant immunotherapy plus chemotherapy and radiotherapy for primary malignant melanoma of the parotid gland (PGMM): A case report

**DOI:** 10.1515/biol-2022-0555

**Published:** 2023-02-09

**Authors:** Qiang Zhao, Zhi-Ke Li, Yan Gui, Dai-Yuan Ma, Guo-Bo Du, Xian-Fu Li

**Affiliations:** Department of Oncology, Affiliated Hospital of North Sichuan Medical College, Shunqing District, Nanchong 637000, Sichuan, China; Department of Oncology, Affiliated Hospital of North Sichuan Medical College, No. 1 Maoyuan South Road, Shunqing District, Nanchong 637000, Sichuan, China

**Keywords:** primary malignant melanoma, parotid gland, adjuvant immunotherapy, combination therapy, radiotherapy

## Abstract

Primary malignant melanoma of the parotid gland (PGMM) is extremely rare, with a poor prognosis. Surgery is the main treatment option followed by adjuvant treatments such as radiotherapy, but which adjuvant treatment to be optimal is still controversial. In this case, a 63-year-old male PGMM patient was first misdiagnosed as a “myoepithelial tumor” and then treated with surgery, postoperative immunotherapy (sintilimab), chemotherapy, and radiotherapy successfully. The progression free survival was more than 19 months without signs of metastasis or recurrence to date. To our best knowledge, this is the first report of postoperative immunotherapy combined with chemotherapy and radiotherapy for PGMM. Our case indicated that combination therapy including surgery, adjuvant immunotherapy (sintilimab) combined with chemotherapy and radiotherapy may be a potential treatment option for PGMM, which needs further research.

## 1 Introduction

Malignant melanoma is a highly invasive form of cancer with a poor prognosis [1]. Most malignant melanomas arise from melanocytes in the skin, but they can also arise from melanocytes in some internal organs and even the central nervous system. In developed countries, the incidence and mortality rates of cutaneous melanoma are approximately (9.3–10.2)/1,000 and (1.2–2.0)/1,000, respectively [2].

Primary malignant melanomas of the head and neck account for 25–30% of all malignant melanomas [3]. Primary malignant melanoma of the parotid gland (PGMM) is extremely rare, accounting for less than 0.7% of parotid malignancies [4], and its prognosis is poor and staging is difficult [5]. Surgery is the first treatment option for PGMM, followed by adjuvant therapies such as radiation, immunotherapy, targeted therapy, and chemotherapy, according to the previous reports [6].

Here, we reported a case of PGMM, which was misdiagnosed as a “myoepithelial tumor” first, and then treated with surgery, postoperative immunotherapy plus chemotherapy combined with radiotherapy successfully.

## 2 Case report

**Figure 1:**
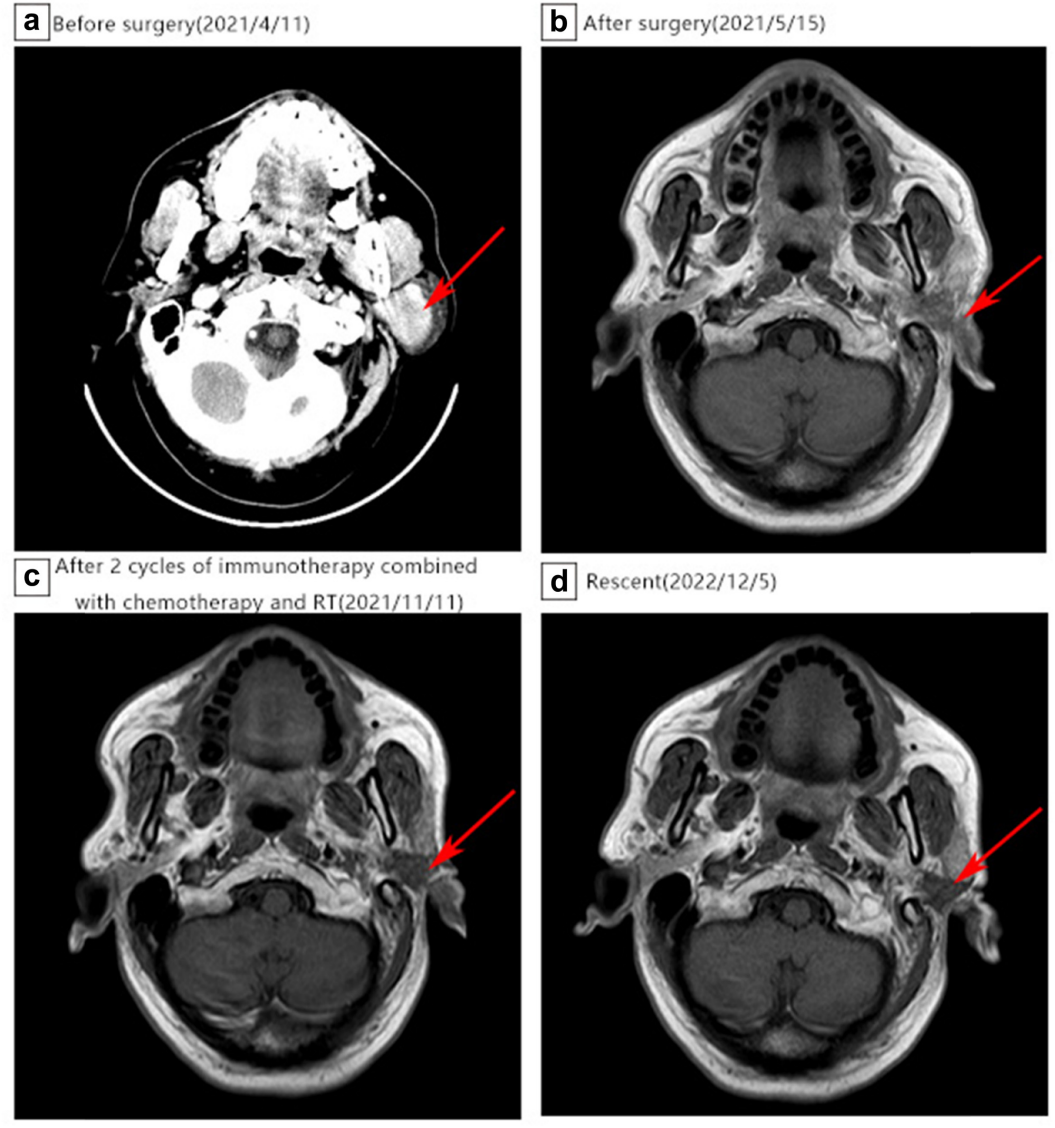
Imaging of the parotid gland. (a) CT scan before surgery, the red arrow shows a left parotid occupancy (on April 11, 2021). (b) Nuclear magnetic resonance (MRI) scan after surgery and before chemotherapy and radiotherapy (RT) (on May 15, 2021). (c) MRI scan after chemotherapy and RT (on November 11, 2021). (d) MRI scan recently (on December 5, 2022).

**Figure 2:**
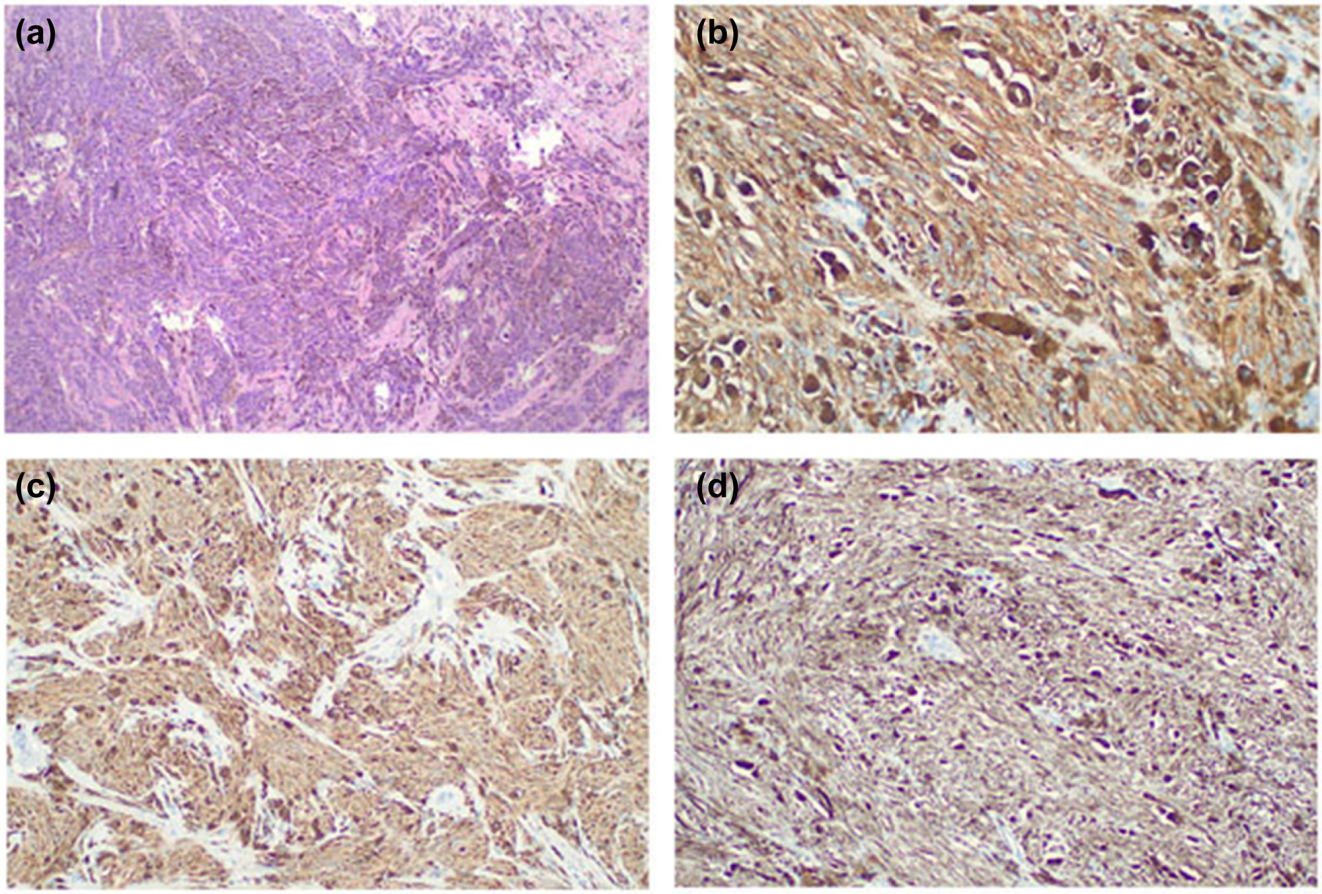
Histopathology of the parotid tumor. (a) The spindle-cell neoplastic cells present variable amounts of melanin (hematoxylin–eosin stain, original magnification, ×40). (b–d) Immunohistochemical staining of the tumor shows strong immunoreactivity in HMB-45, S-100, and SOX10, respectively (immunohistochemical stain, original magnification, ×100).

In January 2021, a 63-year-old male found a painless mass behind his left ear for more than 10 months, with no specific past medical, family, nor personal history. Enhanced computer tomography (CT) of the parotid gland on April 11, 2021 suggested a tumor in the left parotid gland, with a size of approximately 3.0 cm × 2.2 cm (Figure 1a). According to the preoperative images, the clinical diagnosis of a benign tumor was considered. Therefore, the mass was resected as a benign parotid tumor on April 13, 2021. During the operation, the facial nerve was separated, and a 3.0 cm × 2.0 cm × 2.0 cm black mass was found on the deep side of the facial nerve, with clear borderline and tough texture. Intraoperative rapid histopathologic analysis showed a “myoepithelial tumor.” Interestingly, the postoperative histopathologic analysis of two different hospitals (West China Hospital, Sichuan University and Affiliated Hospital of North Sichuan Medical College) was inconsistent with intraoperative analysis. And both immunohistochemistry showed S-100 (+), SOX10 (+), HMB-45 (+), Melan A (+), CK (−), EMA (−). In addition, no EWSR1 gene translocation was found, all supported a malignant melanoma of the left inferior parotid gland (Figure 2), but as the patient refused to accept the gene detection, we could not obtain the gene state of BRAF or MEK. Careful physical examination was performed, and no sign of primary lesions was found. We did not find residual tumor in postoperative MRI (Figure 1b), what else, F-18 FDG PET/CT showed no abnormal tumor metabolic uptake. Therefore, according to immunohistochemical staining and radiographic report, the diagnosis of PGMM was confirmed.

Two months after surgery, the patient returned to our hospital, and received two cycles of immunotherapy combined with chemotherapy (sintilimab 200 mg plus albumin paclitaxel 400 mg, q3w) from June 22, 2021 to July 12, 2021. Then he received radiotherapy for the tumor bed area and the cervical lymph node drainage area with a dose of 48 Gy/12 Fx from August 1, 2021 to August 16, 2021. The MRI right after radiotherapy did not find a tumor (Figure 1c). But after that, he refused further treatment for personal reasons and underwent regular follow-up since then. Up to now, no tumor recurrence or metastasis was found (Figure 1d).


**Informed consent:** Informed consent has been obtained from all individuals included in this study.
**Ethical approval:** The research related to human use has been complied with all the relevant national regulations, institutional policies and in accordance with the tenets of the Helsinki Declaration, and has been approved by the Medical Ethics Committee of Affiliated Hospital of North Sichuan Medical College.

## 3 Discussion

Most parotid malignant melanomas are secondary [5], and primary malignant melanoma is extremely rare. The clinical, pathological, and cytological features of malignant melanoma are variable [7]; therefore, PGMM is extremely easy to be misdiagnosed. Immunohistochemical staining is the gold standard for diagnosis. Based on previous reports, the following rules must be met for the diagnosis of PGMM: (1) the mass must be contained within the parotid gland, (2) parotid masses do not contain metastatic lymph nodes, (3) after careful physical examination, there is no suspected primary lesion of malignant melanoma, and (4) no history or evidence of excision of malignant melanoma or progressive pigmented lesions [8]. The patient in this case met all these conditions. Besides, in our case, immunohistochemistry showed S-100 (+), SOX10 (+), HMB-45 (+), Melan A (+), CK (−), EMA (−). In addition, no EWSR1 gene translocation was found. And no evidence of other primary lesions was found by F-18 FDG PET/CT or physical examination. These results were confirmed in two different large hospitals, thus the diagnosis of PGMM was reliable.

The first treatment options for PGMM are parotidectomy and cervical lymph node dissection [9]. But, the parotidectomy approach is still controversial. Some researchers have shown that total parotidectomy does not improve life expectancy but leads to poorer quality of life, so radical parotidectomy should be avoided if possible [4]. However, Wertz et al. supported complete parotidectomy, they concluded that total parotidectomy may have a lower recurrence rate than superficial lobectomy [10]. Because of the high incidence of occult metastatic lymph nodes, at least selective lymph node dissection should be performed even when no lymph node metastasis to be found in the neck, which can reduce the probability of recurrence while reducing the incidence of adverse events and improving the quality of life [9].

Local recurrence occurs in about 30–50% of patients who do not receive adjuvant therapy after surgery, and the 10-year overall survival (OS) is about 25–40% [11]. If patients have high risk factors, such as tumor invasion of facial nerve, the prognosis is even worse. Postoperative adjuvant therapy can reduce the risk of postoperative recurrence, and the reported main postoperative therapies are radiotherapy, immunotherapy, targeted therapy, interferon, and chemotherapy [6]. The efficacy of chemotherapy, radiotherapy, and interferon for PGMM is controversial [4], and it is still unknown which adjuvant therapy is the best [6].

In recent years, more and more studies have demonstrated the therapeutic benefit of immunotherapy in malignant melanoma, which is gradually becoming the most promising treatment modality for malignant melanoma [12]. In the NCT00636168 trial, ipilimumab, an anti-cytotoxic T-lymphocyte-associated antigen 4 (CTLA 4) monoclonal antibody, has been reported to be effective in malignant melanoma. The Food and Drug Administration had approved ipilimumab in 2015 as an adjuvant therapy for stage III melanoma patients with high-risk recurrence, but its use was limited by autoimmune toxicity [13]. As adjuvant immunotherapy, the anti-programmed death 1 (PD-1) inhibitor nivolumab has a higher recurrence-free survival (RFS) and better tolerance compared to ipilimumab [14]. Adjuvant pembrolizumab (a PD-1 inhibitor) for resectable stage III malignant melanoma is effective, with 1-year RFS rate of 75% [15].

Sintilimab is a human immunoglobulin G4 monoclonal antibody which can bind to PD-1, block PD-1 interaction with its ligand, and help restore the endogenous antitumor response of T-cells. Compared with nivolumab and pembrolizumab, sintilimab has a similar antitumor effect, better safety profile, and significant economic advantage [16]. Sintilimab is mainly used to treat lymphoma, lung cancer, liver cancer, stomach cancer, and esophageal carcinoma [17]. Sintilimab combined with anti-CTLA-4 is also effective in advanced melanoma [18].

In our case, considering that the patient did not receive radical resection of the parotid gland or lymph node dissection of the neck, and without genetic testing, the gene state of BRAF and MEK was unknown, thus there is no evidence for targeted therapy. He started radiotherapy with a dose of 48 Gy/12 Fx after two cycles of postoperative adjuvant immunotherapy in combination with chemotherapy (sintilimab 200 mg plus albumin paclitaxel 400 mg, q3w). The biological equivalent dose of radiotherapy was higher than previous reports [19].

Most studies about PGMM are case reports. One study showed that approximately 89.7% of PGMM patients underwent parotidectomy, 52.5% of PGMM patients underwent cervical lymph node dissection, and 25% of PGMM patients underwent postoperative radiotherapy [20]. Distant metastases occurred in two-thirds of PGMM patients during follow-up, with a mean follow-up time of 7.1 months [20]. An article reported a PGMM patient who underwent total parotidectomy with facial nerve preservation and cervical lymph node dissection, followed by interferon α-2a treatment, with a progression-free survival (PFS) of more than 6 years [21], indicating that long-term survival may be possible for patients who received immunotherapy.

This is the first report of postoperative sintilimab combined with chemotherapy and radiotherapy for PGMM. The prognosis of primary and metastatic parotid malignant melanoma is both poor, with an average OS of less than 2 years [5]. In our case, the patient’s PFS was more than 19 months with no signs of recurrence or metastasis. The therapeutic value of sintilimab in PGMM needs to be evaluated in further clinical trials. However, based on the outcome of this patient, adjuvant immunotherapy of sintilimab combined with chemotherapy and radiotherapy may be a potential adjuvant treatment option for PGMM.

## 4 Conclusion

In conclusion, this case may suggest that surgery, adjuvant immunotherapy of sintilimab combined with chemotherapy and radiotherapy may be a potential treatment for PGMM, which needs further research.
